# Molecular Basis of Influence of A501X Mutations in Penicillin-Binding Protein 2 of *Neisseria gonorrhoeae* Strain 35/02 on Ceftriaxone Resistance

**DOI:** 10.3390/ijms25158260

**Published:** 2024-07-29

**Authors:** Alexandra V. Krivitskaya, Maria S. Kuryshkina, Maria Y. Eremina, Ivan V. Smirnov, Maria G. Khrenova

**Affiliations:** 1 Bach Institute of Biochemistry, Federal Research Centre “Fundamentals of Biotechnology”, Russian Academy of Sciences, 119071 Moscow, Russia; al_krivickaya@mail.ru; 2 Chemistry Department, Lomonosov Moscow State University, 119991 Moscow, Russia; mariia.kuryshkina@chemistry.msu.ru (M.S.K.); smirnov@ibch.ru (I.V.S.); 3 Emanuel Institute of Biochemical Physics, Russian Academy of Sciences, 119334 Moscow, Russia; 4 Biology Department, Lomonosov Moscow State University, 119234 Moscow, Russia; 5 Shemyakin-Ovchinnikov Institute of Bioorganic Chemistry, Russian Academy of Sciences, 117997 Moscow, Russia

**Keywords:** antibiotic resistance, QM/MM, molecular dynamics, ceftriaxone, penicillin-binding protein (PBP2)

## Abstract

The increase in the resistance of mutant strains of *Neisseria gonorrhoeae* to the antibiotic ceftriaxone is pronounced in the decrease in the second-order acylation rate constant, k_2_/K_S_, by penicillin-binding protein 2 (PBP2). These changes can be caused by both the decrease in the acylation rate constant, k_2_, and the weakening of the binding affinity, i.e., an increase in the substrate constant, K_S_. A501X mutations in PBP2 affect second-order acylation rate constants. The PBP2^A501V^ variant exhibits a higher k_2_/K_S_ value, whereas for PBP2^A501R^ and PBP2^A501P^ variants, these values are lower. We performed molecular dynamic simulations with both classical and QM/MM potentials to model both acylation energy profiles and conformational dynamics of four PBP2 variants to explain the origin of k_2_/K_S_ changes. The acylation reaction occurs in two elementary steps, specifically, a nucleophilic attack by the oxygen atom of the Ser310 residue and C–N bond cleavage in the β-lactam ring accompanied by the elimination of the leaving group of ceftriaxone. The energy barrier of the first step increases for PBP2 variants with a decrease in the observed k_2_/K_S_ value. Submicrosecond classic molecular dynamic trajectories with subsequent cluster analysis reveal that the conformation of the β_3_–β_4_ loop switches from open to closed and its flexibility decreases for PBP2 variants with a lower k_2_/K_S_ value. Thus, the experimentally observed decrease in the k_2_/K_S_ in A501X variants of PBP2 occurs due to both the decrease in the acylation rate constant, k_2_, and the increase in K_S_.

## 1. Introduction

The last line of defense against gonorrhea is the β-lactam antibiotic ceftriaxone, which belongs to the cephalosporin group. This disease is caused by the *Neisseria gonorrhoeae* bacterium [1], and its treatment by ceftriaxone assumes covalent binding to the PBP and inhibition of the crosslinking of the peptidoglycan multilayers [2,3]. Antibiotic resistance frequently occurs due to mutations in the *penA* gene encoding PBP2, which is the main β-lactam antimicrobial target of gonococci [4]. Variants of the *penA* gene can carry a single point mutation close to the active site and contain up to 70 amino acid residues [5,6,7,8].

Several new antibiotics are currently undergoing clinical evaluation for the treatment of gonorrhea [9,10,11,12]. Namely, the Global Antibiotic Research & Development Partnership (GARDP), in collaboration with Innoviva Specialty Therapeutics, has reported positive phase 3 trial results for a first-in-class antibiotic, zoliflodacin [13]. If approved, zoliflodacin would represent the first new antibiotic for gonorrhea treatment in decades. However, at present, ceftriaxone monotherapy remains the sole available treatment in many countries [14]. An emerging problem in ceftriaxone therapy is the accumulation of mutations in a gene encoding PBP2 of the bacterium *N. gonorrhoeae* that leads to the development of resistance to the antibiotic [15,16].

It was shown from the structural side that there is a correlation between the conformation and dynamics of the β_3_–β_4_ loop (residues 498–514) of PBP2 and the extent of ceftriaxone resistance. Wild-type PBP2 exhibits a dynamic exchange between two distinct conformational states: a low-affinity state characterized by an extended β_3_–β_4_ loop conformation and a high-affinity state featuring an inward β_3_–β_4_ loop conformation [17]. Mutations found in PBP2 from the ceftriaxone-resistant strain H041 of *N. gonorrhoeae* are shown to confer resistance by either destabilizing the inward β_3_–β_4_ loop conformation or stabilizing the extended β_3_–β_4_ loop conformation [18,19]. These alterations favor the low-affinity drug-binding state, reducing the proportion of target molecules in the high-affinity drug-binding state. 

The recent identification of fully cefixime- and ceftriaxone-resistant clinical isolates with an A501P mutation underscores the significance of mutations at the Ala501 residue in resistance augmentation. The potential of Ala501 mutations in PBP2 from strain 35/02 (PBP2^35/02^) to enhance resistance to expanded-spectrum cephalosporins has been systematically studied [20]. Only five Ala501 substitutions (A501V, A501T, A501P, A501R, and A501S) preserve transpeptidase function. Structural insight suggests that increased rigidity in the active site region serves as a mechanism for cephalosporin resistance mediated by Ala501 mutations in PBP2. In particular, mutations at Ala501 to valine and threonine are associated with increased minimum inhibitory concentrations (MICs) of extended-spectrum cephalosporins. There is not always a direct correlation between the acylation rate and MIC, as other factors such as the extent of PBP2 inhibition required for cell death can also influence MIC. The second-order rate constant (k_2_/K_S_) is often used to assess enzyme efficiency. Here, it is a ratio of the rate constant k_2_ of covalent PBP2–ceftriaxone complex formation to the thermodynamic ceftriaxone–PBP2 dissociation constant, K_S_. k_2_/K_S_ values are known for some PBP2 variants. The k_2_/K_S_ value for PBP2^A501V^ is 1.8-fold higher, and the k_2_/K_S_ values for PBP2^A501R^ and PBP2^A501P^ are 1.8- and 45-fold lower, respectively, compared with PBP2^35/02^. Individual catalytic parameters, acylation rate constant, k_2_, and binding affinity, K_S_, are unknown for these systems.

**Figure 1:**
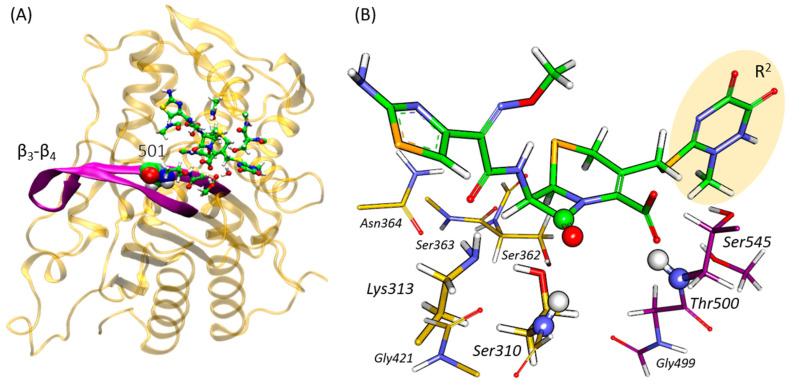
(**A**) The structure of PBP2^35/02^ (yellow ribbons) with the β_3_–β_4_ loop colored purple (residues 498–518); the QM part in the QM/MM MD simulations is shown in the balls and sticks representation. Ala501 that is substituted with other residues is highlighted by van der Waals spheres. (**B**) The QM subsystem. The fragment of the antibiotic ceftriaxone leaving during the reaction is in the yellow oval, the spheres indicate the atoms of the carbonyl group of ceftriaxone, and the atoms form the oxyanion hole (NH fragments of Thr500 and Ser310). The color code is oxygen—red, nitrogen—blue, sulfur—yellow, and hydrogen—white. The color code for carbon atoms is green for ceftriaxone, purple for fragments of the β_3_–β_4_ loop and yellow for the rest of the protein.

Previously, molecular modeling studies have demonstrated that mutations leading to increased resistance impact not only the conformation of the β_3_–β_4_ loop but also affect the active site region [21]. The majority of mutations associated with resistance affect the active site area, altering the acylation reaction mechanism [21]. An oxyanion hole is formed by NH fragments of Thr500 and Ser310 and is responsible for the binding and activation of the carbonyl fragment of ceftriaxone in the active site of PBP2^35/02^ [21,22]. Residue 501 is located near the oxyanion hole, indicating that amino acid substitutions are likely to impact not only the mobility of the loop but also the enzymatic reaction in the active site (Figure 1). 

This study investigates the impact of a mutation at residue 501 in the transpeptidase PBP2 of the strain 35/02 of *Neisseria gonorrhoeae* on the mobility of the β_3_–β_4_ loop and the acylation mechanism of ceftriaxone in the active site. We performed molecular dynamic (MD) simulations with the combined quantum mechanics/molecular mechanics (QM/MM) potentials to compare the dynamic behavior of enzyme-substrate (ES) complexes and reconstruct energy profiles of elementary steps corresponding to acylation. Classical MD runs of sub-microsecond trajectories were comprehensively analyzed to discriminate different conformational states of the β_3_–β_4_ loop.

## 2. Results and Discussion

### 2.1. Molecular Mechanism of Ceftriaxone Acylation by Ser310 of PBP2

**Figure 2:**
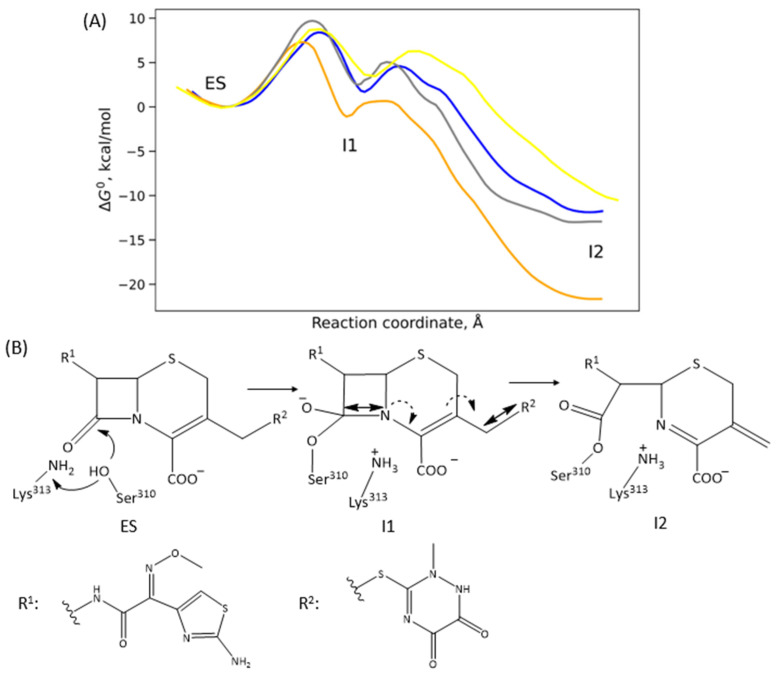
(**A**) Gibbs energy profile of elementary steps of acylation reaction in active site of PBP2^A501V^ (orange), PBP2^A501R^ (yellow), PBP2^A501P^ (gray), and PBP2^35/02^ (blue). (**B**) Molecular mechanism of reaction: plain black arrows are reaction coordinates; dashed black arrows depict redistribution of electron pairs.

The Gibbs energy profiles of the elementary steps of the acylation reaction in an active site of PBP2 variants and a schematic illustration of the reaction are presented in Figure 2. The QM/MM MD structures of all states along the reaction path are presented in the Supporting Information (Figure S1) and deposited at ZENODO (a link is available in the Supporting Information). The enzyme–substrate complex dynamics were analyzed for all considered systems. We found no pronounced difference correlated with kinetic properties in the key interatomic distances, including hydrogen bonds in the oxyanion hole and the distance of the nucleophilic attack (Figure S2). More complex combinations of geometry criteria can likely provide insight into the origin of hydrolytic activity differences.

The first step of acylation is the nucleophilic attack of Ser310 side-chain oxygen on the carbonyl carbon of the β-lactam ring of ceftriaxone accompanied by the proton transfer from the Ser310 OH group to the amino group of Lys313. The reaction coordinate of this elementary step is the sum of distances d(N_Lys313_…H_Ser310_) and d(O_Ser310_…C), and it changes from ~4.5 Å at the enzyme–substrate complex to ~2.5 Å in the I1 state. The ES minimum has almost the same reaction coordinate values. Reaction coordinate values in the transition state and at the intermediate (I1) vary depending on the particular variant of PBP2. The complex with PBP2^A501V^ has an earlier transition state (TS1) and an intermediate with the larger reaction coordinate value. For others, reaction coordinates at both of these points are larger. The Gibbs energy barrier for the PBP2^A501^-containing system is 8.4 kcal/mol. For PBP2^A501V^, the energy barrier is 7.3 kcal/mol, and it is consistent with the increase in second-order rate constants for acylation compared to PBP2^A501^. For PBP2^A501R^ and PBP2^A501P^ variants exhibiting decreases in k_2_/K_S_, the energy barriers are 8.8 and 9.7 kcal/mol, respectively, which is consistent with the extent of the rate constant decrease. 

The second step comprises simultaneous C–N bond cleavage of the β-lactam ring and elimination of the leaving group, R^2^, of the antibiotic. The latter occurs due to the cleavage of the C–S bond accompanied by the transfer of the excess negative charge to the leaving group. The reaction coordinate at this step is the sum of the distances d(C…N) and d(C…S). The second step occurs with the low energy barrier for all considered systems and leads to considerable stabilization of the system due to the energy decrease. This step is practically irreversible as the R^2^ fragments diffuse into the bulk after elimination from the protein.

Thus, the chemical step of the reaction, at least to some extent, is responsible for changes in the k_2_/K_S_-caused amino acid substitutions at residue 501. This is in line with variations in the k_2_/K_S_ for variants of PBP2 from different strains [21]. 

### 2.2. Dynamic Behavior of the β_3_–β_4_ Loop

**Figure 3:**
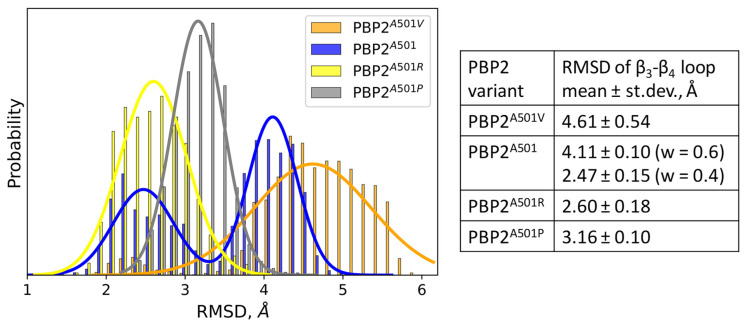
RMSD distribution plot for β_3_–β_4_ loop of variants of PBP2 and corresponding mean values and standard deviation of normal distributions. For PBP2^A501^, weight of each mode is in parenthesis.

Classical MD simulations were performed for the *apo*-form of all considered PBP2 variants. The RMSD was calculated for the β_3_–β_4_ loop (Figure 3). The alignment was performed over the backbone of the entire protein selected for all PBP2 variants, whereas the RMSD was calculated only for the β_3_–β_4_ loop. This allowed us to define both the changes in the conformation of the loop and its position relative to the rest of the protein. The PBP2^A501^ variant demonstrates bimodal distribution, whereas the three other variants are characterized by single-mode distribution. The RMSD distribution of the most efficient (with the largest k_2_/K_S_) PBP2^A501V^ variant overlaps with the mode of distribution of PBP2^A501^ with larger RMSD values. In contrast, the RMSD distributions for the less efficient PBP2^A501R^ and PBP2^A501P^ are shifted to smaller values and overlap with another mode of PBP2^A501^. Importantly, the widths of the distributions decrease with the decrease in the k_2_/Ks value as the measure of loop flexibility. Thus, the β_3_–β_4_ loop of PBP2^A501P^ is the least flexible and that of PBP2^A501V^ is the most flexible. The bimodal distribution of the RMSD of PBP2^A501^ might be an indication of two types of conformations.

**Figure 4:**
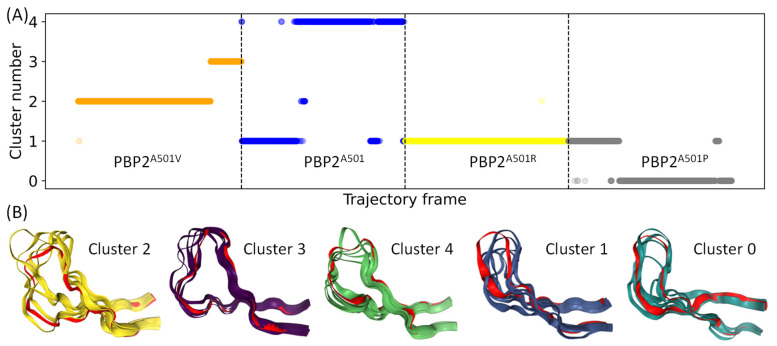
EnGens analysis of PBP2^A501^ and its variants. (**A**) The timeline view of 500 ns trajectories with the assignment of each frame to a particular cluster with respect to the β_3_–β_4_ loop conformation. The dots represent MD frames and the colors correspond to the PBP2 variant (PBP2^A501V^—orange; PBP2^A501^—blue; PBP2^A501R^—yellow; PBP2^A501P^—gray). The vertical dashed lines depict the endings and beginnings of the trajectories. (**B**) A cartoon representation of 5 random states belonging to a cluster and the state that is the closest to the cluster center is colored red.

We combined all MD trajectories to perform a joint analysis of conformations of the β_3_–β_4_ loop using clusterization. We started with a set of dihedrals describing the backbone of the loop and then reduced the space to 14 principal components. The entire set of conformations is described by five clusters (Figure 4). The results of clusterization are shown in Figure 4. The dynamic behavior of the PBP2^A501P^ variant with the highest antibiotic resistance demonstrates a closed, slightly moving β_3_–β_4_ loop pressed to the protein. PBP2^A501V^ with the highest k_2_/K_S_ value is characterized by an open, externally oriented, and labile β_3_–β_4_ loop. The PBP2^A501^ variant is characterized by two different types of conformations that are in line with the bimodal distribution of the RMSD values (Figure 3). One of them shares the same cluster with the PBP2^A501R^ variant, which can be attributed to the closed states. The other cluster of PBP2^A501^ corresponds to the open state.

**Figure 5:**
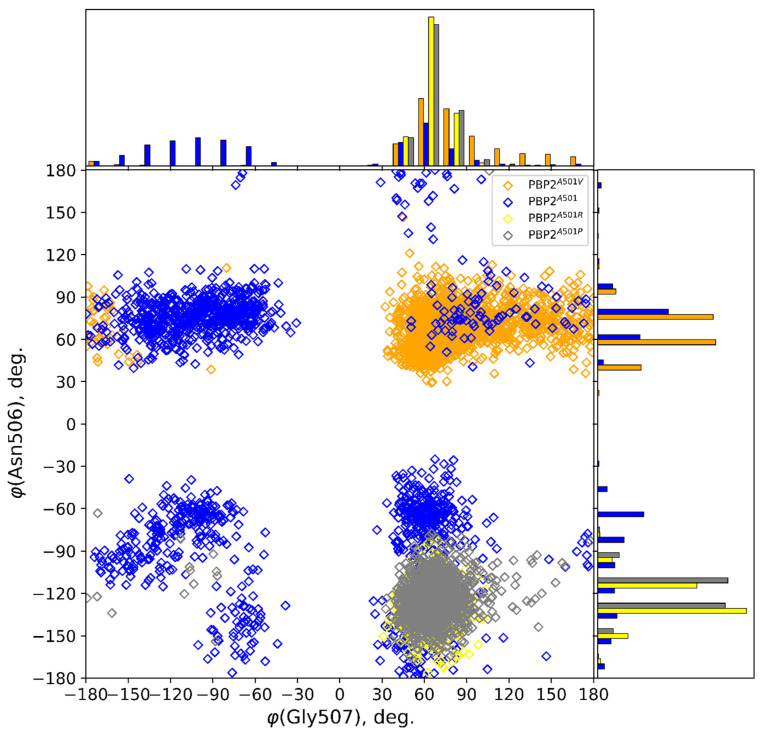
Distribution of φ(Gly507) and φ(Asn506) over MD trajectories for PBP2 variants.

The following analysis of clusters was performed to determine the individual geometry parameters of the β_3_–β_4_ loop mostly contributing to variations in the conformations (Figure 5). The main contributions to PCs are torsions of the main chain of residues Leu504, Asn506, Gly507, Val510, and Asp511. For the PBP2^A501P^ and PBP2^A501R^ variants with a lower acylation efficiency and a less flexible loop in closed conformation, the φ dihedral (C–N–C_α_–C of the backbone) of Asn506 varies in the range of −60°…−180°. For PBP2^A501V^, the same dihedral ranges between 40° and 120°. The distribution of φ(Asn506) is bimodal for the PBP2^A501^ variant, similar to the clusterization results. Another important parameter is φ(Gly507). For all PBP2 variants with mutations, distributions are unimodal with φ(Gly507) = 20°…180°. For PBP2^A501^, φ(Gly507) is also bimodal. Two populations dominate, with φ(Asn506) = −30°…−180°, φ(Gly507) = 30°…90°, φ(Asn506) = 30°…100°, and φ(Gly507) = −50°…−180°. Thus, we can conclude that the mutations at the 501 residue affect the conformation and flexibility of the β_3_–β_4_ loop, which is mostly pronounced in conformations of the Asn506 and Gly507 backbones. 

## 3. Materials and Methods

The complex of the PBP2^35/02^ *apo*-form was obtained from the crystal structure PDB ID: 6VBL with 1.9 Å resolution [18]. During crystallization, the protein was truncated, residues 283–297 were removed, and a new Gly297 was introduced to connect the two dangling ends. Therefore, there were reconstructed truncated residues according to the primary amino acid sequence from UniProt (Figure S1A). There was no structure for the complex of PBP2^35/02^ with ceftriaxone or with any other antibiotic. To construct the enzyme–substrate complex, an additional study was carried out with the available crystal structures of PBP2 from the wild-type strain FA19 and PBP2 from the mutant strain H041 [21]. The crystal structure of the acyl–enzyme complex of PBP2^FA19^ with ceftriaxone was obtained from the crystal structure PDB ID: 6P54, and PBP2^H041^ with ceftriaxone was obtained from the crystal structure PDB ID: 6VBD. Then, ceftriaxone was recovered to its initial state in the PBP2^H041^–ceftriaxone structure, and its position was determined using molecular dynamics calculations. Then, the reaction mechanism was calculated from the acyl–enzyme structure to the enzyme–substrate structure. This approach allows one to avoid errors when manually constructing an enzyme–substrate complex. The resulting enzyme–substrate complexes PBP2^FA19^–ceftriaxone and PBP2^H041^–ceftriaxone were analyzed using QM/MM MD. The analysis showed different substrate positions in the active site of PBP2 from these strains due to the appearance of the G545S substitution. In PBP2 from the mutant strains, Ser545 interacts with ceftriaxone carboxylate. This interaction shifts the position of the substrate closer to the exit from the binding pocket in PBP2 from the mutant strains. Since PBP2 from strain 35/02 also contains the substitution G545S, ceftriaxone was placed in the active site of PBP2^35/02^, similar to that in the ceftriaxone complex with PBP2^H041^ [18]. Hydrogen atoms were added using the Reduce program [23] in such a way that the protonated forms of the amino acids with ionogenic groups corresponded to a neutral pH, except for the catalytic residue Lys313, which remained in the neutral form as a proton acceptor of the OH group of the catalytic residue Ser310 during the nucleophilic attack. For mutant forms of PBP2^35/02^, a point substitution of A501X was performed, and the considered variants were Ala501, Pro501, Val501, and Arg501. ES complexes were solvated in the rectangular water box so that the distance from the protein to the cell border exceeded 12 Å and was neutralized. Initial equilibration of ES complexes was performed for 20 ns (Figure S1B). CHARMM36 [24,25] force field parameters were utilized for the protein, TIP3P [26] for water molecules, and CGenFF [27,28,29] for ceftriaxone in all classic molecular dynamic simulations and in QM/MM MD simulations for the MM subsystem. All MD trajectories were calculated at T = 300 K and p = 1 atm with a 1 fs integration time step. The calculations were performed using the NAMD program package (version 3.0 Alpha 11, Urbana, IL, USA) [30]. Preliminary 20 ns runs were performed to equilibrate the systems. Equilibration was controlled by the RMSD graph calculated over all heavy atoms (Figure S3B). For the conformational analysis of the β_3_–β_4_ loop, 500 ns trajectories were computed for each system. 

The EnGens service [31] was utilized to analyze the MD trajectories. We selected torsion angles of the main chain of the β_3_–β_4_ loop to discriminate between different states. Next, we performed principal component analysis (PCA) to reduce the dimension; the top 14 principal components (PCs) were selected for the following analysis. The number of PCs was chosen such that the variance was greater than 80%. Subsequent clustering was carried out using the KMeans method. The optimal number of clusters was determined by the sum of the distances within the clusters, and we chose the number of clusters where this value decreased to a greater extent. The representative structure of the cluster was the point closest to the center of the cluster.

QM/MM MD calculations were performed in the NAMD program package, combined with TeraChem (version 1.93P, Los Altos Hills, CA, USA) [32], and integrated by a special script [33]. The cutoff distance for point charges of the MM subsystem contributing to the QM Hamiltonian was 12 Å. The quantum subsystem included the substrate molecule, the catalytic residues Lys313 and Ser310, the amino acid residues forming the oxyanion hole, the residues interacting with substrate or catalytic residues (Ser362, Asn364, Thr500, and Ser545), and two solvation water molecules. The quantum subsystem was described at the Kohn–Sham DFT level with the PBE0 hybrid functional [34] with D3 dispersion correction [35] and a 6-31G** basis set. 

The Gibbs energy profiles for each elementary step along the reaction pathway were calculated using the umbrella sampling approach [36,37]. The sets of 5–10 ps runs were performed with harmonic potentials centered at different values of reaction coordinates. The force constant of the harmonic potential ½·K·(ξ − ξ_0_)^2^ was usually set to 40 kcal/mol/Å^2^, and additional trajectories with K = 80–120 kcal/mol/Å^2^ in transition state regions were calculated in several runs. Harmonic potentials were centered every 0.2 Å along the reaction coordinates. The MD trajectories of the PBP2 enzyme and its mutant forms were combined using both the weighted histogram analysis method (WHAM) and umbrella integration (UI). The quality of distributions was monitored by the overlap and consistency of Gibbs energy profiles obtained with both WHAM and UI methods.

## 4. Conclusions

We performed molecular dynamics simulations with classical and QM/MM potentials to reveal the origin of changes in the second-order ceftriaxone acylation rate constant, k_2_/K_S_, observed in A501X variants of PBP2. QM(PBE0-D3/6-31G**)/MM molecular dynamics reveal that the acylation reaction occurs via two elementary steps. The first step is the nucleophilic attack of the carbonyl carbon atom of ceftriaxone by the oxygen atom of the side chain of the Ser310 residue. The second step is C–N bond cleavage in the β-lactam ring accompanied by the elimination of the leaving group of ceftriaxone. The energy barrier of the first step increases for PBP2 variants with smaller k_2_/K_S_ values. It is already known that the conformation and flexibility of the β_3_–β_4_ loop are responsible for the antibiotic binding efficiency. We calculated submicrosecond classical MD trajectories followed by principal component and cluster analysis to discriminate between conformations of this loop on A501X variants of PBP2. The β_3_–β_4_ loop switches from an open to a closed state, and its flexibility decreases for PBP2 variants with lower k_2_/K_S_ values. Thus, the decrease in the k_2_/K_S_ in A501X variants of PBP2 occurs due to both the decrease in the acylation rate constant, k_2_, and the lowering of the binding affinity, i.e., an increase in K_S_.

## Data Availability

Full-atom molecular models of all considered systems and representative frames from classical MD trajectory clusterization are available at ZENODO https://doi.org/10.5281/zenodo.11660179 (accessed on 24 July 2024).

## Supplementary Materials

The supporting information can be downloaded at: https://www.mdpi.com/article/10.3390/ijms25158260/s1.

## References

[1] Hanke J., Brett D., Zastrow I., Aydin A., Delbrück S., Lehmann G., Luft F., Reich J., Bork P. (1999). Alternative splicing of human genes: More the rule than the exception?. Trends Genet..

[2] Sauvage E., Kerff F., Terrak M., Ayala J.A., Charlier P. (2008). The penicillin-binding proteins: Structure and role in peptidoglycan biosynthesis. FEMS Microbiol. Rev..

[3] Mucsi Z., Chass G.A., Ábrányi-Balogh P., Jójárt B., Fang D.-C., Ramirez-Cuesta A.J., Viskolcz B., Csizmadia I.G. (2013). Penicillin’s catalytic mechanism revealed by inelastic neutrons and quantum chemical theory. Phys. Chem. Chem. Phys..

[4] López-Argüello S., Montaner M., Mármol-Salvador A., Velázquez-Escudero A., Docobo-Pérez F., Oliver A., Moya B. (2023). Penicillin-Binding Protein Occupancy Dataset for 18 β-Lactams and 4 β-Lactamase Inhibitors in Neisseria gonorrhoeae. Microbiol. Spectr..

[5] Camara J., Serra J., Ayats J., Bastida T., Carnicer-Pont D., Andreu A., Ardanuy C. (2012). Molecular characterization of two high-level ceftriaxone-resistant Neisseria gonorrhoeae isolates detected in Catalonia, Spain. J. Antimicrob. Chemother..

[6] Shaskolskiy B., Dementieva E., Kandinov I., Filippova M., Petrova N., Plakhova X., Chestkov A., Kubanov A., Deryabin D., Gryadunov D. (2019). Resistance of Neisseria gonorrhoeae isolates to beta-lactam antibiotics (benzylpenicillin and ceftriaxone) in Russia, 2015–2017. PLoS ONE.

[7] Lahra M.M., Martin I., Demczuk W., Jennison A.V., Lee K.-I., Nakayama S.-I., Lefebvre B., Longtin J., Ward A., Mulvey M.R. (2018). Cooperative Recognition of Internationally Disseminated Ceftriaxone-Resistant Neisseria gonorrhoeae Strain. Emerg. Infect. Dis..

[8] Tomberg J., Unemo M., Davies C., Nicholas R.A. (2010). Molecular and Structural Analysis of Mosaic Variants of Penicillin-Binding Protein 2 Conferring Decreased Susceptibility to Expanded-Spectrum Cephalosporins in Neisseria gonorrhoeae: Role of Epistatic Mutations. Biochemistry.

[9] Chen M.Y., McNulty A., Avery A., Whiley D., Tabrizi S.N., Hardy D., Das A.F., Nenninger A., Fairley C.K., Hocking J.S. (2019). Solithromycin versus ceftriaxone plus azithromycin for the treatment of uncomplicated genital gonorrhoea (SOLITAIRE-U): A randomised phase 3 non-inferiority trial. Lancet Infect. Dis..

[10] Fernandes P., Craft J.C. (2019). Phase 3 trial of treating gonorrhoea with solithromycin. Lancet Infect. Dis..

[11] de Vries H.J.C., Schim-van der Loeff M.F. (2019). Solithromycin for the treatment of drug-resistant gonorrhoea. Lancet Infect. Dis..

[12] Goytia M., Thompson S.T., Jordan S.V.L., King K.A. (2021). Antimicrobial Resistance Profiles of Human Commensal Neisseria Species. Antibiotics.

[13] Bradford P.A., Miller A.A., O’Donnell J., Mueller J.P. (2020). Zoliflodacin: An Oral Spiropyrimidinetrione Antibiotic for the Treatment of Neisseria gonorrheae, Including Multi-Drug-Resistant Isolates. ACS Infect. Dis..

[14] Bell S.F.E., Ware R.S., Lewis D.A., Lahra M.M., Whiley D.M. (2023). Antimicrobial susceptibility assays for Neisseria gonorrhoeae: A proof-of-principle population-based retrospective analysis. Lancet Microbe.

[15] Tomberg J., Unemo M., Ohnishi M., Davies C., Nicholas R.A. (2013). Identification of Amino Acids Conferring High-Level Resistance to Expanded-Spectrum Cephalosporins in the penA Gene from Neisseria gonorrhoeae Strain H041. Antimicrob. Agents Chemother..

[16] Spratt B.G. (1988). Hybrid penicillin-binding proteins in penicillin-resistant strains of Neisseria gonorrhoeae. Nature.

[17] Fenton B.A., Tomberg J., Sciandra C.A., Nicholas R.A., Davies C., Zhou P. (2021). Mutations in PBP2 from ceftriaxone-resistant Neisseria gonorrhoeae alter the dynamics of the β3–β4 loop to favor a low-affinity drug-binding state. J. Biol. Chem..

[18] Singh A., Turner J.M., Tomberg J., Fedarovich A., Unemo M., Nicholas R.A., Davies C. (2020). Mutations in penicillin-binding protein 2 from cephalosporin-resistant Neisseria gonorrhoeae hinder ceftriaxone acylation by restricting protein dynamics. J. Biol. Chem..

[19] Ohnishi M., Golparian D., Shimuta K., Saika T., Hoshina S., Iwasaku K., Nakayama S., Kitawaki J., Unemo M. (2011). Is Neisseria gonorrhoeae Initiating a Future Era of Untreatable Gonorrhea?: Detailed Characterization of the First Strain with High-Level Resistance to Ceftriaxone. Antimicrob. Agents Chemother..

[20] Tomberg J., Fedarovich A., Vincent L.R., Jerse A.E., Unemo M., Davies C., Nicholas R.A. (2017). Alanine 501 Mutations in Penicillin-Binding Protein 2 from Neisseria gonorrhoeae: Structure, Mechanism, and Effects on Cephalosporin Resistance and Biological Fitness. Biochemistry.

[21] Krivitskaya A.V., Khrenova M.G. (2022). Evolution of Ceftriaxone Resistance of Penicillin-Binding Proteins 2 Revealed by Molecular Modeling. Int. J. Mol. Sci..

[22] Krivitskaya A.V., Khrenova M.G. (2022). Molecular modeling of ceftriaxone activation in the active sites of penicillin-binding proteins 2. Russ. Chem. Bull..

[23] Word J.M., Lovell S.C., Richardson J.S., Richardson D.C. (1999). Asparagine and glutamine: Using hydrogen atom contacts in the choice of side-chain amide orientation. J. Mol. Biol..

[24] Denning E.J., Priyakumar U.D., Nilsson L., Mackerell A.D. (2011). Impact of 2′-hydroxyl sampling on the conformational properties of RNA: Update of the CHARMM all-atom additive force field for RNA. J. Comput. Chem..

[25] Best R.B., Zhu X., Shim J., Lopes P.E.M., Mittal J., Feig M., MacKerell A.D. (2012). Optimization of the Additive CHARMM All-Atom Protein Force Field Targeting Improved Sampling of the Backbone ϕ, ψ and Side-Chain χ_1_ and χ_2_ Dihedral Angles. J. Chem. Theory Comput..

[26] Jorgensen W.L., Chandrasekhar J., Madura J.D., Impey R.W., Klein M.L. (1983). Comparison of simple potential functions for simulating liquid water. J. Chem. Phys..

[27] Vanommeslaeghe K., Hatcher E., Acharya C., Kundu S., Zhong S., Shim J., Darian E., Guvench O., Lopes P., Vorobyov I. (2010). CHARMM general force field (CGenFF): A force field for drug-like molecules compatible with the CHARMM all-atom additive biological force fields. J. Comput. Chem..

[28] Vanommeslaeghe K., MacKerell A.D. (2012). Automation of the CHARMM General Force Field (CGenFF) I: Bond Perception and Atom Typing. J. Chem. Inf. Model..

[29] Vanommeslaeghe K., Raman E.P., MacKerell A.D. (2012). Automation of the CHARMM General Force Field (CGenFF) II: Assignment of Bonded Parameters and Partial Atomic Charges. J. Chem. Inf. Model..

[30] Phillips J.C., Hardy D.J., Maia J.D.C., Stone J.E., Ribeiro J.V., Bernardi R.C., Buch R., Fiorin G., Hénin J., Jiang W. (2020). Scalable molecular dynamics on CPU and GPU architectures with NAMD. J. Chem. Phys..

[31] Conev A., Rigo M.M., Devaurs D., Fonseca A.F., Kalavadwala H., de Freitas M.V., Clementi C., Zanatta G., Antunes D.A., Kavraki L.E. (2023). EnGens: A computational framework for generation and analysis of representative protein conformational ensembles. Brief. Bioinform..

[32] Seritan S., Bannwarth C., Fales B.S., Hohenstein E.G., Isborn C.M., Kokkila-Schumacher S.I.L., Li X., Liu F., Luehr N., Snyder J.W. (2021). TeraChem: A graphical processing unit-accelerated electronic structure package for large-scale ab initio molecular dynamics. WIREs Comput. Mol. Sci..

[33] Melo M.C.R., Bernardi R.C., Rudack T., Scheurer M., Riplinger C., Phillips J.C., Maia J.D.C., Rocha G.B., Ribeiro J.V., Stone J.E. (2018). NAMD goes quantum: An integrative suite for hybrid simulations. Nat. Methods.

[34] Adamo C., Barone V. (1999). Toward reliable density functional methods without adjustable parameters: The PBE0 model. J. Chem. Phys..

[35] Grimme S., Antony J., Ehrlich S., Krieg H. (2010). A consistent and accurate ab initio parametrization of density functional dispersion correction (DFT-D) for the 94 elements H-Pu. J. Chem. Phys..

[36] Kästner J., Thiel W. (2005). Bridging the gap between thermodynamic integration and umbrella sampling provides a novel analysis method: “Umbrella integration”. J. Chem. Phys..

[37] Kästner J. (2011). Umbrella sampling. Wiley Interdiscip. Rev. Comput. Mol. Sci..

