# Supplementary material for: Molecular Basis of Influence of A501X Mutations in Penicillin-Binding Protein 2 of Neisseria gonorrhoeae Strain 35/02 on Ceftriaxone Resistance
Source: Int J Mol Sci. 2024 Jul 29;25(15):8260. doi: 10.3390/ijms25158260 (PMC11312080; doi:10.3390/ijms25158260)
Supplement: Supplementary file 1 [file ijms-25-08260-s001.zip › ijms-3083515-supplementary.pdf]

Supporting Information for

Molecular basis of influence of A501X mutations in penicillin-binding protein 2 of *Neisseria gonorrhoeae* strain 35/02 on ceftriaxone resistance

Alexandra V. Krivitskaya [1], Maria S. Kuryshkina [2,3], Maria Y. Eremina [4], Ivan V. Smirnov [2,5] and Maria G. Khrenova[1,2*]

[1] Bach Institute of Biochemistry, Federal Research Centre "Fundamentals of Biotechnology" of the Russian Academy of Sciences, 119071, Moscow, Russia
[2] Chemistry Department, Lomonosov Moscow State University, 119991 Moscow, Russia
[3] Emanuel Institute of Biochemical Physics, Russian Academy of Sciences, 119334, Moscow, Russia
[4] Biology Department, Lomonosov Moscow State University, 119234 Moscow, Russia
[5] Shemyakin-Ovchinnikov Institute of Bioorganic Chemistry of the Russian Academy of Sciences, 117997 Moscow, Russia
***** Correspondence: Corresponding author: Prof. Maria G. Khrenova, E-mail: mkhrenovamg@my.msu.ru

PDB structures are deposited at the ZENODO and can be found at https://doi.org/10.5281/zenodo.12786163. Among them are representative frames obtained in QM/MM MD simulations for all considered PBP2 variants with ceftriaxone for the stationary point regions (the entire systems and QM parts). The corresponding states are aligned on Figure S1. Representative frames from classical MD trajectories from each cluster (Figure 4) are also deposited.

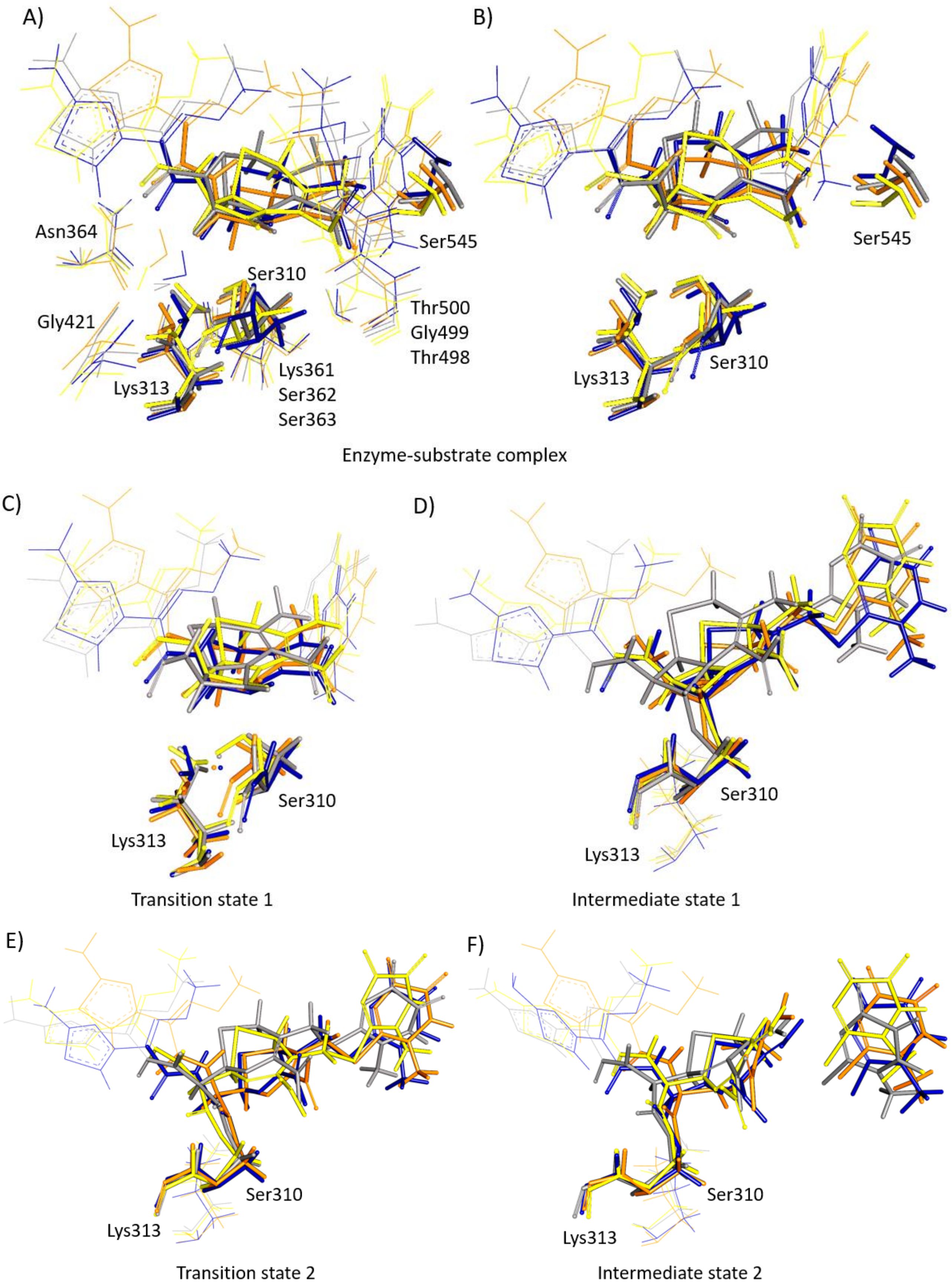

Figure S1. A-B) Superposition of QM parts of all the considered systems for enzyme-substrate state (A-B), transition state 1 (C), intermediate state 1(D), transition state 2 (E) and intermediate state 2 (F). Superposition on C_α atoms of Ser310, Ser362, C_β of Asn364 and C_γ of Lys313.The figures of QM part complete structures of all the systems under consideration looks loaded. Therefore, some of the residues were removed from the figures to make it easier to perceive. The color code: PBP^{A501} - blue, PBP^{A501V} - orange, PBP^{A501R} – yellow, PBP^{A501P} – grey.

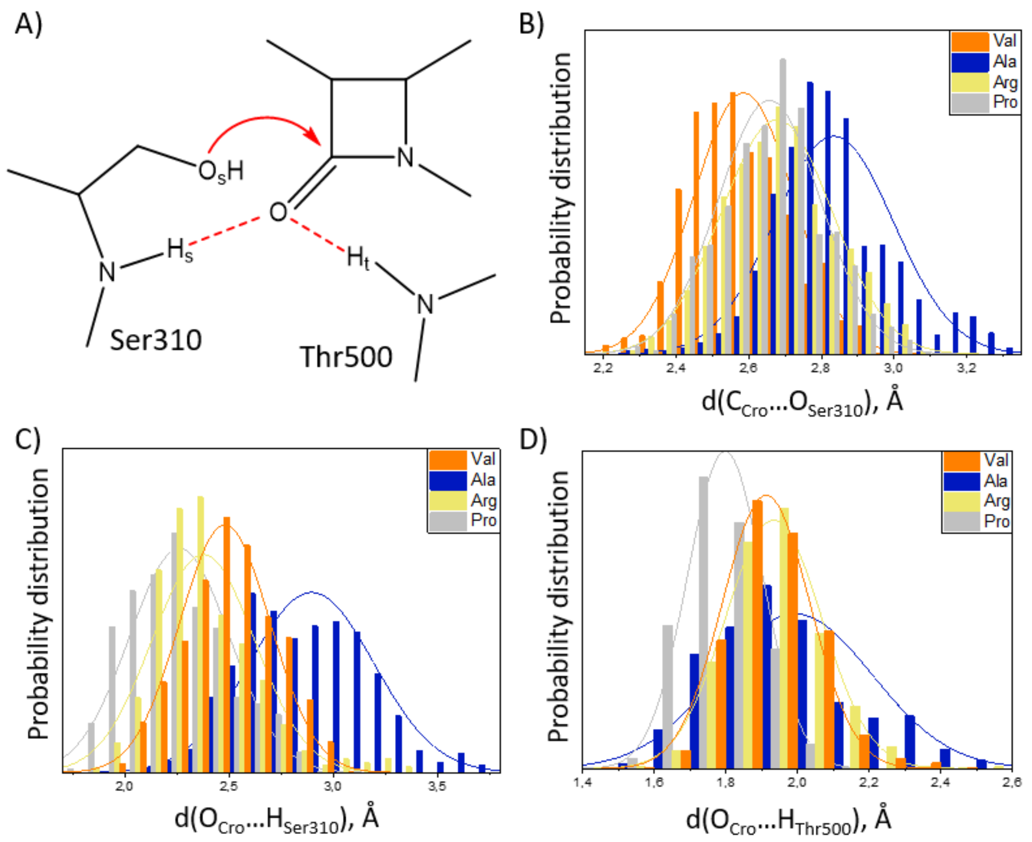

Figure S2. A) Composition of the oxyanion hole. B) Nucleophilic attack distance distribution, d(C_{Cro}…O_{Ser310}); C-D) hydrogen bond distributions, d(O_{Cro}…H_{Ser310}) and d(O_{Cro}…H_{Thr500}). All distribution are obtained from 5 ps QM/MM MD trajectory.

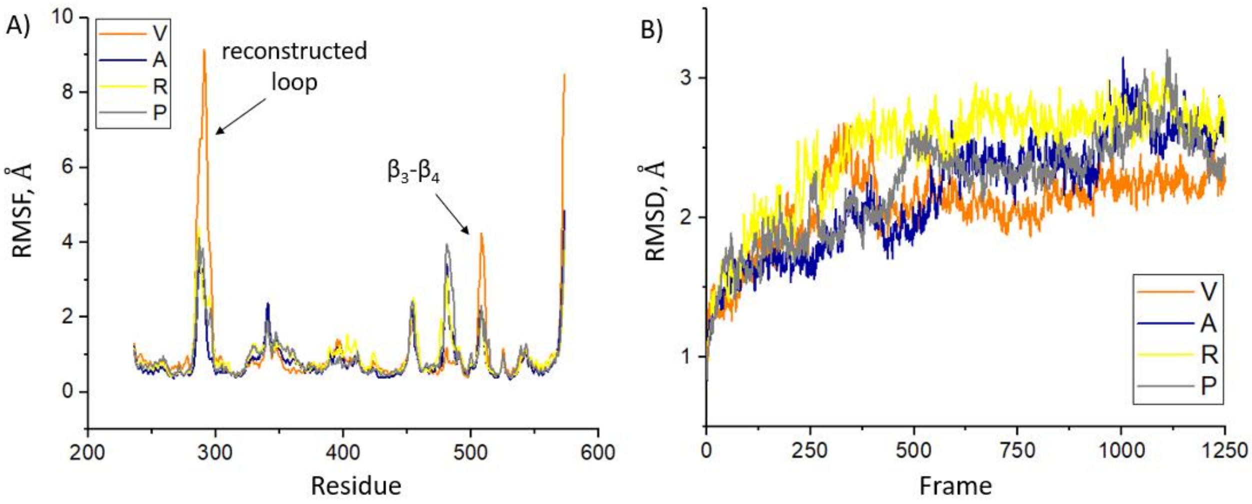

Figure S3. (A) RFSM for 500 ns trajectory of A501X Variants of *apo*-form of PBP2; (B) RMSD of 20 ns trajectory of cefrriaxone-PBP2 complexes. The color code: PBP2^{A501}- blue, PBP2^{A501V}- orange, PBP2^{A501R}- yellow, PBP2^{A501P}- grey.
